# Immunohaematological reference values in human immunodeficiency virus-negative adolescent and adults in rural northern Tanzania

**DOI:** 10.1186/1471-2334-9-1

**Published:** 2009-01-13

**Authors:** Bernard J Ngowi, Sayoki G Mfinanga, Johan N Bruun, Odd Morkve

**Affiliations:** 1 Haydom Lutheran Hospital, Mbulu District, Manyara Region, Tanzania; 2 National Institute for Medical Research, Haydom Research Station, Tanzania; 3 National Institute for Medical Research, Muhimbili Medical Research Centre, Dar es Salaam, Tanzania; 4 Department of Infectious Diseases, Ulleval University Hospital, Oslo, Norway; 5 Department of Internal Medicine B, University Hospital of North Norway, Tromsø, Norway; 6 Centre for International Health University of Bergen, Bergen, Norway; 7 Department of Thoracic Diseases, Haukeland University Hospital, Bergen, Norway

## Abstract

**Background:**

The amount of CD4 T cells is used for monitoring HIV progression and improvement, and to make decisions to start antiretroviral therapy and prophylactic drugs for opportunistic infections. The aim of this study was to determine normal reference values for CD4 T cells, lymphocytes, leucocytes and haemoglobin level in healthy, HIV negative adolescents and adults in rural northern Tanzania.

**Methods:**

A cross sectional study was conducted from September 2006 to March 2007 in rural northern Tanzania. Participants were recruited from voluntary HIV counselling and testing clinics. Patients were counselled for HIV test and those who consented were tested for HIV. Clinical screening was done, and blood samples were collected for CD4 T cell counts and complete blood cell counts.

**Results:**

We enrolled 102 participants, forty two (41.2%) males and 60 (58.8%) females. The mean age was 32.6 ± 95% CI 30.2–35.0. The mean absolute CD4 T cell count was 745.8 ± 95% CI 695.5–796.3, absolute CD8 T cells 504.6 ± 95% CI 461.7–547.5, absolute leukocyte count 5.1 ± 95% CI 4.8–5.4, absolute lymphocyte count 1.8 ± 95% CI 1.7–1.9, and haemoglobin level 13.2 ± 95% CI 12.7–13.7. Females had significantly higher mean absolute CD4 T cell count (p = 0.008), mean absolute CD8 T cell count (p = 0.009) and significantly lower mean haemoglobin level than males (p = 0.003)

**Conclusion:**

Immunohaematological values found in this study were different from standard values for western countries. Females had significantly higher mean CD4 T cell counts and lower mean haemoglobin levels than males. This raises the issue of the appropriateness of the present reference values and guidelines for monitoring HIV/AIDS patients in Tanzania.

## Background

Immunohaematological indices such as leukocytes, lymphocytes and their subsets play a major role in both cellular and humoral types of immunity. CD4 T cells are the lymphocytes used for monitoring progression of HIV infection, and they are also used as a surrogate marker for the improvement of HIV patients after initiation of anti retro-viral (ARV) therapy [1-5]. Furthermore, CD4 T cell level determines when to start or stop prophylactic drugs for opportunistic infections [2,3,6]. Management of HIV patients include proper monitoring, irrespective of ARV treatment. This monitoring can be done clinically by means of the WHO clinical staging, but more reliably by measuring CD4 T cells and viral load [2,3]. Immunohaematological variations have been reported in various studies, showing association with sex, [7,8] geographical location, race, altitude and diet [7,9-12]. Other reasons for variations are pregnancy, age, [13,14] exercise, cormobid conditions and diurnal variation [15-17], in addition to variations caused by methodological differences. Several studies have shown significant variations of CD4 T cells within African populations and in Africans compared with the values established for Europe and North America [12,18-26]. This justifies the importance of establishing local immunological reference values for the local African population. We report on immunohaematological indices in HIV negative, healthy individuals in rural Tanzania.

## Methods

### Study design, setting and population

A cross sectional study was conducted in three Divisions: Dongobesh in Mbulu District, Basotu in Hanang District (both in Manyara Region) and Nduguti in Iramba District (Singida Region). The total population in these three divisions is about 250,000 [27]. In each division there are mobile HIV voluntary counselling and testing (VCT) services which are conducted once per month in each ward in the respective divisions. The VCT services are under Haydom Lutheran Hospital which is owned by The Evangelical Lutheran Church in Tanzania, Mbulu Diocese.

### Study subjects

Study subjects were adolescents and adults who attended HIV-VCT from September 2006 to March 2007. Eligible subjects were those aged 10 years and above and who agreed to participate in the study. Those who consented were asked for blood samples for repeat HIV test, CD4 T cell and CD8 T cell counts, and complete blood cell count (CBC). Blood was collected in ethylene diamine-tetra-acetic acid (EDTA) tubes. The same samples were used for repeat HIV test, CBC, CD4 T cell and CD8 T cell counts. Samples were collected in the morning from 9.00 AM to 12.00 noon and kept at room temperature and transferred to Haydom hospital for analysis within the same day.

Subjects were interviewed, using a structured questionnaire, and screened for symptoms such as fever, cough and weight loss to rule out any recent and/or current infections. Blood slide for malaria was done for all participants, in addition to a physical examination, including measurement of height and weight.

The following categories were excluded: pregnant (1), smokers (8), patients receiving medical treatment, individuals with history of recent or current cormobid conditions, chronic alcoholism and moderate and severe malnourishment (BMI< 17.5 Kg/m^2^) [28] (9), patients with malaria (7) and individuals who tested positive for HIV antibody (2).

### Laboratory procedure

#### HIV test

HIV test was done using 2 different rapid antibody tests, Determine HIV-1/2 (Abbott laboratories, Abbott Park, IL, USA) and Capillus HIV-1/2 (Trinity Biotech, Bray, Co Wicklow, Ireland). Concordant positive results were interpreted as positive for HIV antibody. Discordant results were interpreted as inconclusive and the samples were sent to the regional hospital for confirmatory test using ELISA; Enzygnost anti-HIV 1+2 Plus ELISA (Behring, Marburg, Germany) and Well-coenzyme HIV recombinant ELISA (Murex, Dartford, England) [29,30].

#### Haematological analysis

Complete blood cell counts were done using Sysmex Kx-21 (Sysmex Corporation; Kobe Japan). The machine automatically dilutes a whole-blood sample, lyses, counts and gives a printout result of absolute numbers of leucocytes (expressed as number of cells × [10^9^] per liter), erythrocytes (number of cells × [10^12^] per liter), platelets (number of cells × [10^9] ^per liter), lymphocytes (number of cells × [10^9^] per liter), mononuclear cells (number of cells × [10^9^] per liter), granulocytes (number of cells × [10^9^] per liter) and haemoglobin (grams per decilitre). The quality and accuracy of the technique and the machine was assessed every six months.

#### CD4 T cell counts

CD4 T cells were analyzed using a FACSCount flow cytometer (Becton Dickinson Immunocytometry Systems, San Jose, Calif.). In brief, 50 μl of whole blood was mixed and incubated at room temperature for 20 min with 20 μl of aCD4 and aCD8. Red blood cells were then lysed by adding 450 μl of fluorescence-activated cell sorter lysing solution (Becton Dickinson Immunocytometry Systems). The tubes were incubated at room temperature for 10 min, and then analyzed with the FACSCounts's Cell Quest software (Becton Dickinson Immunocytometry Systems) within six hours. By using quality control (Multicheck; Becton Dickinson Immunocytometry Systems), the accuracy of the technique was assessed every 6 months.

#### Screening for Malnutrition

All participants were assessed for malnutrition using body mass index (BMI). Normal nutritional status was defined as BMI>18.5 Kg/m^2^, mild malnutrition as BMI of 17.5–18.4 Kg/m^2^, moderate malnutrition as BMI of 16–17.4 Kg/m^2 ^and severe malnutrition as BMI of < 16 Kg/m^2^[28].

#### Data management and Statistical analysis

Completed questionnaires were coded by numbers and double entered in a computer software Epi-data version 13.1. Cross-checking and data cleaning was done. The data was then transferred to SPSS version 13 for analysis[31] Chi-squire test was used to explore bivariate associations for categorical variables. Where appropriate we used student (t) test. Logistic regression was used to explore the association of socio-demographics and other variables with CD4 T cells. Odds ratio (OR) with 95% confidence intervals (CI) was used.

#### Ethical considerations

The protocol was approved by the Medical Research Coordinating Committee of the Ministry of Health and Social Welfare, Tanzania. Oral informed consent was obtained from the patients prior to enrolment. For those below the age of 18 years permission was sought from parents or caretakers.

Those found to have HIV infection on the repeat HIV test were excluded from the study and referred to the HIV care and treatment clinic for further management. HIV/AIDS diagnosis, treatment and monitoring were given free of charge according to Tanzanian national policy.

## Results

### Baseline characteristics of the study participants

Initially 129 persons volunteered to participate in the study. After screening for current or recent comorbid conditions, pregnancy and smoking we remained with 102 participants. Forty two (40%) were males and 60 (59%) were females. The mean age was 32.6 ± 95% CI 30.2–35.0. For males the mean age was 35.2 ± 95% CI 31.5–50.5 and for females 30.9 ± 95% CI 49.5–68.5.

### Immunohaematological parameters and sex

The results for the immunohaematological values are given in table 1 and 2

**Table 1:** Arithmetic median, 2.5^th^–97.5^th ^percentile, mean and 95% CI of the mean CD4 T cells, CD8 T cells, CD4/CD8 ratio and haemoglobin level

Sex	n	Median	2.5^th^–97.5^th ^percentile	Mean ± SD	95% CI of the Mean	P value
**Absolute CD4 T cells**						
Males	42	596.5	291.2–1278.9	665.6 ± 246.8	588.7–742.5	t = 2.7, df = 89, p = 0.008
Females	60	764.5	288.5–1406.8	802.0 ± 250.2	737.4–866.7	
Total	102	722.5	312.2–1367.6	745.9 ± 256.6	695.5–796.3	
**Absolute CD8 T cells**						
Males	42	417	145.9–977.3	438.2 ± 208.4	373.4–503.0	t = 2.7, df = 90, p = 0.009
Females	60	513	171.7–1101.7	551.0 ± 215.4	495.4–606.7	
Total	102	472	168.2–996.8	504.6 ± 218.6	461.7–547.5	
**CD4/CD8 T cells ratio**						
Males	42	1.6	1.0–3.7	1.6 ± 0.5	1.5–1.9	t = 1.2, df = 61, p = 0.2
Females	60	1.5	1.1–2.4	1.5 ± 0.3	1.4–1.6	
Total	102	1.5	1.1–2.5	1.6 ± 0.4	1.5–1.6	
**Haemoglobin level**						
Males	42	14.8	7.0–18.0	14.1 ± 2.7	13.3–14.9	t = 3.1, df = 68, p = 0.003
Females	60	13.0	7.5–15.4	12.6 ± 1.9	12.0–13.1	

Total	102	13.5	7.1–17.4	13.6 ± 2.4	12.7–13.7	

Reference value are reported in percentile

**Table 2:** Arithmetic median, 2.5^th^–97.5^th ^percentile mean and 95% CI of the mean leukocytes, lymphocytes and nutritional status

Sex	n	Median	2.5^th^–97.5^th ^percentile	Mean ± SD	95% CI of the Mean	P value
**Absolute leukocytes**						
Males	42	4.7	2.3–8.8	4.9 ± 1.6	4.4–5.4	t = 1.2, df = 91, p = 0.2
Females	60	5.2	2.5–9.2	5.3 ± 1.7	4.8–5.7	
Total	102	5.0	2.5–9.0	5.1 ± 1.6	4.8–5.4	
**Absolute lymphocytes**						
Males	42	1.5	0.6–3.2	1.6 ± 4.9	1.5–1.8	t = 0.3, df = 92, p = 0.8
Females	60	1.8	0.3–4.7	1.9 ± 5.3	1.8–2.2	
Total	102	1.7	0.6–3.5	1.8 ± 5.1	1.7–1.9	
**Nutritional status given as BMI in Kg/m**^2^						
Males	42	21.9	17.6–34.5	23.3 ± 3.9	22.0–24.5	t = 0.4, df = 71, p = 0.7
Females	60	23.6	17.9–32.9	23.6 ± 2.9	22.8–24.3	

Total	102	22.9	17.9–32.9	23.4 ± 3.4	22.8–24.1	

Reference value are reported in percentile

Reference values are reported in percentiles in these tables

Females had higher mean absolute leukocyte counts and lymphocyte counts than males (table 2). Females also had significantly higher mean absolute CD4 T cells, (t = 2.7 df = 89, p = 0.008) and higher mean absolute CD8 T cells (t = 2.7, df = 90, p = 0.009) than males. For the haemoglobin level females had significantly lower mean haemoglobin level than males, (t = 3.2, df = 68, p = 0.03). Table 3 shows the difference between males and females with lower than normal and normal ranges of the immunohaematological values. There was a statistical significance among participants with CD4 T cells <500 cells/mm^3^, where males had significantly lower CD4 T cells than females (p = 0.01, OR 4.4, 95% CI 1.3–15). There was no statistically significant difference between males and females having lower than normal haemoglobin levels (X^2 ^= 1.1, df = 1, p = 0.2), lower than normal leucocytes (X^2 ^= 0.1, df = 1, p = 0.7) and lower than normal lymphocytes (X^2 ^= 2.5, df = 1, p = 0.1)

**Table 3:** Males and females with lower than normal and normal values of CD4 T cells, leukocytes, lymphocytes, haemoglobin level and body mass index

Parameter	Males		Females		Total	P-value
		
**CD4 T cells in cells/mm**^3^	n	%	n	%		
<500	10	23.8	4	6.7	14	0.01
>500	32	76.2	56	93.3	88	
Total	42	100	60	100	102	
Leukocytes in cells/litre						
<4.0 × 10^9^	10	23.3	16	26.6	26	0.7
>4.0 × 10^9^	32	76.2	44	73.4	76	
Total	42	100	60	100	102	
Lymphocytes in cells/litre						
<1.0 × 10^9^	8	19.0	5	8.3	13	0.1
> 1.0 × 10^9^	34	81.0	55	91.7	89	
Total	42	100	60	100	102	
Haemoglobin level in g/dl						
<10.0	4	9.5	10	16.7	14	0.1
>10.0	38	90.5	50	83.3	88	
Total	42	100	60	100	102	
**Body mass index in kg/m**^2^						
17.0–18.4	1	2.4	2	3.3	3	0.7
>18.5	41	97.6	58	96.7	99	
Total	42	100	60	100	102	

### Immunohaematological parameters and nutritional status

On nutritional status assessment using BMI 3 (3%) of the participants had mild malnutrition (BMI 17.5–18.4) and 99 (97%) had normal nutritional status (BMI>18.5). The mean BMI for the participants was 23.4 ± 95% CI 22.8–24.1; Mean BMI for males was 23.3 ± 95% CI 22.0–24.5 and for females 23.6 ± 95% CI 22.8–24.3 (Table 2). There was no statistically significant difference between males and females in nutritional status (X^2 ^= 0.07, df = 1, p = 0.8). There was no significant association between nutritional status and any of the immunohaematological parameters, neither by bivariate nor by multivariate analysis.

### Immunohaematological parameters and age

There was no statistically significant association between age and any of the immunohaematological parameters.

## Discussion

Absolute CD4 T cell counts and haemoglobin level are the most important parameters for monitoring progression of HIV to AIDS and also the improvement after initiation of antiretroviral therapy (ARV). Tanzania is scaling up ARV for people living with HIV/AIDS, and CD4 T cell counts and haemoglobin level are the recommended parameters for monitoring these patients [1-3]. In our study we found that females have significantly higher counts of absolute CD4 T cells and absolute CD8 T cells than males, and also higher mean absolute leukocyte and lymphocyte counts. Our results are similar to those reported in other studies in African settings [7,8,12,32]. More important, our findings are different from the standard values established for Europe and Northern America [33]. There are also several other studies done in Africa and Asia that report different values for CD4 T cell levels compared to standard values for Western countries [12,16,22]. Studies done in Tanzania and Cameroon have reported higher CD4 T cell counts as compared to Ethiopia, Botswana and Uganda [12,34-37], whereas some studies have demonstrated higher CD4 T cells among Ugandans and Kenyans than the values known for North America, Europe and Asia [32]. These variations in CD4 T cells have been shown to be associated with ethnicity, gender, diet, geographical area, as well as being dependent of genetic and environmental factors [7,9-12]. The value of the CD4 T cell counts obtained in our study is lower than those reported for Ethiopian and the Dutch counterparts [34,38]

In our study significant proportion of males had lower mean absolute CD4 T cell and absolute CD8 T cell counts than females. This finding is similar to a study done in Tanzania to determine gender difference in CD4 T cells, which showed that males were more likely to have CD4 T cells <500 cells/mm^3^[35]. In addition to the variation of CD4 T cells with the above factors, the CD4 T cells variation with sex might complicate the management of people living with HIV/AIDS, especially in decision making for starting prophylaxis for opportunistic infections and ARV.

Females had significantly lower haemoglobin levels than males in our study. This is similar to findings in North America, Europe and Asia. Overall we found low haemoglobin levels compared to European values and the standard reference haemoglobin values. This can be partly explained by low dietary intake of food rich in iron and vitamins, which is the commonest causes of low haemoglobin levels in Tanzania; In addition infections such as malaria and worm infestations are well known cause of low haemoglobin levels [39]. The values obtained for haemoglobin levels in this study is lower than the currently value used in Tanzania. This shows that a significant proportion of this population is anaemic, though we did not explore the causes of anaemia in our study. Overall the mean absolute leukocyte, lymphocyte and haemoglobin levels are lower than the standard reference values used, similar to other studies in Africa [12,32] The causes of these variations are unknown, though genetic factors, diet, altitude and environmental factors such as infections can partly explain the differences[12,32]

The strengths of our study are that data were collected from an African population living in a rural area in the same environment as the patients. Blood samples were collected and analysed within the same day. Also, we used the currently recommended methods in Tanzania for enumerating the CD4 T cells and white blood cells, (FACSCount and automated haematology machine Sysmex Kx 21). A limitation of our study is that we have a small sample size. Also, though we ruled out obvious comorbid conditions by clinical screening and laboratory investigations such as HIV test and blood slides for malaria, we might had missed conditions such as latent tuberculosis infection, asymptomatic syphilis and also HIV infection during the window period because our HIV test can not pick up HIV antibodies before 3 months. We might also erroneously have excluded some of the participants because of over-reporting of clinical symptoms used as screening tool for inclusion.

## Conclusion

Immunohaematological values found in this study were different from the standard values for the western countries. Females had significantly higher mean CD4 T cell and CD8 T cell counts and lower mean haemoglobin levels than males. The important issue raised by our study, and supported by other studies is: what are the consequences of using western reference values and guidelines for monitoring HIV/AIDS patients if the normal values for the CD4 T cells are different in African context, and females have higher CD4 T cells and CD8 T cells than males? What are the implications for the initiation and monitoring of patients on ARV and prophylactic drugs for opportunistic infections? We believe it is time for a review of the reference values for CD4 T cells and other immunohaematological parameters used to monitor patients, and prepare guidelines according to local African findings.

## Competing interests

The authors declare that they have no competing interests.

## Authors' contributions

All authors contributed to the paper, all authors conceived the study. BJN conducted the study. SGM, JNB and OM supervised the research. BJN and OM analysed the data. All authors helped to conceptualise ideas and interpret the findings. BJN prepared the draft, other authors helped to review and finalise the manuscript

## Pre-publication history

The pre-publication history for this paper can be accessed here:

http://www.biomedcentral.com/1471-2334/9/1/prepub
